# Supplementary material for: Subgenic Pol II interactomes identify region‐specific transcription elongation regulators
Source: Mol Syst Biol. 2017 Jan 2;13(1):900. doi: 10.15252/msb.20167279 (PMC5293154; doi:10.15252/msb.20167279)
Supplement: Supplementary file 1 — Expanded View Figures PDF [file MSB-13-900-s001.pdf]

Expanded View Figures

Figure EV1. Distribution of summed MS1 intensities.

A–D The distribution of summed MS1 intensities from all peptides corresponding to each protein identified in each mass spectrometry run are plotted. The distribution of MS1 intensities is plotted for all nine samples (triplicate 5′ IPs, triplicate 3′ IPs, and triplicate mock IPs). Also shown is a table displaying the mean and standard deviation for the number of unique proteins identified from the triplicate IPs. The distributions are plotted for (A) raw MS1 intensities, (B) mean normalized (see Materials and Methods) intensities, (C) mean normalized intensities filtered for proteins present in all three samples from at least one condition, and (D) mean normalized intensities filtered for proteins present in all three samples from at least one condition with missing values (i.e. proteins present in one condition but not the other) imputed (see Materials and Methods). The mean and variance are shown in the table next to the distributions represent the mean and variance from averaging the triplicate datasets. *t*-tests to test for differences in the mean and *F*-tests to test for differences in the variance for each dataset are also shown. These data demonstrate that both raw and normalized datasets do not vary in their variances as *F*-tests between the datasets are not significant. The 5′ and 3′ IPs show highly similar mean MS1 intensity values and distributions in the raw data. The mock IPs display a mean shifted toward larger MS1 intensities. Thus, to allow for comparison between 5′, 3′ IPs, and mock IPs, the data were mean normalized (as described in the Materials and Methods) to center the distributions of the datasets around zero, allowing the samples to be compared to one another. As the variance was not significantly different between any of the datasets, no adjustment of the variance was made. After mean normalization, the MS1 intensity distributions and variances are all similar, allowing direct comparison of protein enrichment within samples as well as *t*-tests between each protein in each sample. Note that dataset mean and variance values do not differ between samples until imputation of missing values to allow for statistical analysis between samples.

▶

© 2017 The Authors

A

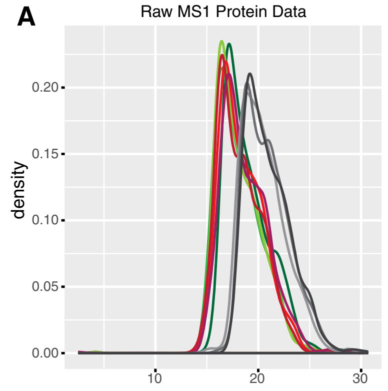

	GFP 5'	RFP 3'	Mock
Unique proteins identified (Mean ± SD)	808 ± 22	854 ± 8	711 ± 39

	GFP 5'	RFP 3'	Mock
Mean	18.41	18.46	20.81
Variance	4.82	4.74	4.67

	GFP 5' vs mock	RFP 3' vs mock	GFP 5' vs RFP 3'
T test, p value (mean similarity)	$2.2X10^{-16}$	$2.2X10^{-16}$	0.5138
F test, pvalue (variance similarity)	0.8278	0.6703	0.8301

B

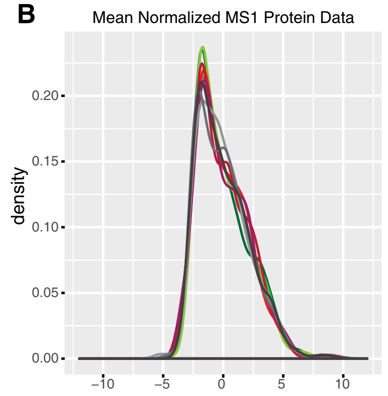

	GFP 5'	RFP 3'	Mock
Mean	$4.45x10^{-7}$	$3.01x10^{-7}$	$3.86x10^{-7}$
Variance	4.82	4.74	4.67

	GFP 5' vs mock	RFP 3' vs mock	GFP 5' vs RFP 3'
T test, p value (mean similarity)	0.9809	0.8996	0.8761
F test, pvalue (variance similarity)	0.9171	0.6794	0.7483

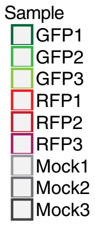

C

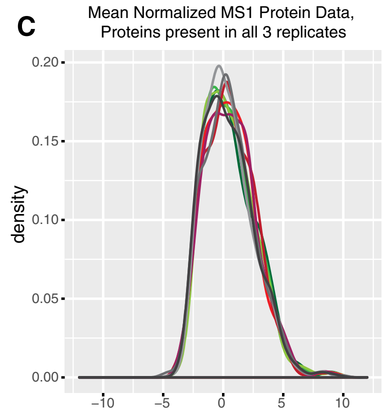

	GFP 5'	RFP 3'	Mock
Mean	0.56	0.58	0.46
Variance	4.54	4.45	4.50

	GFP 5' vs mock	RFP 3' vs mock	GFP 5' vs RFP 3'
T test, p value (mean similarity)	0.2631	0.06078	0.447
F test, pvalue (variance similarity)	0.9959	0.8081	0.801

D

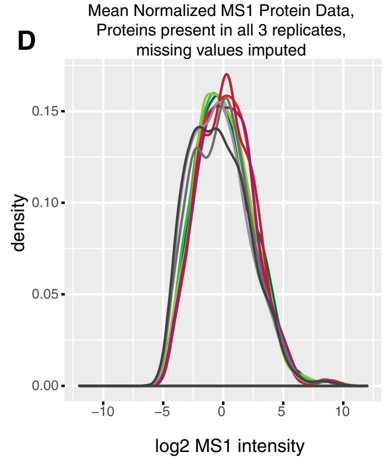

	GFP 5'	RFP 3'	Mock
Mean	0.07	0.24	-0.28
Variance	5.69	5.30	5.99

	GFP 5' vs mock	RFP 3' vs mock	GFP 5' vs RFP 3'
T test, p value (mean similarity)	0.0050	$1.79x10^{-5}$	0.1422
F test, pvalue (variance similarity)	0.4459	0.09665	0.3684

Figure EV1.

© 2017 The Authors

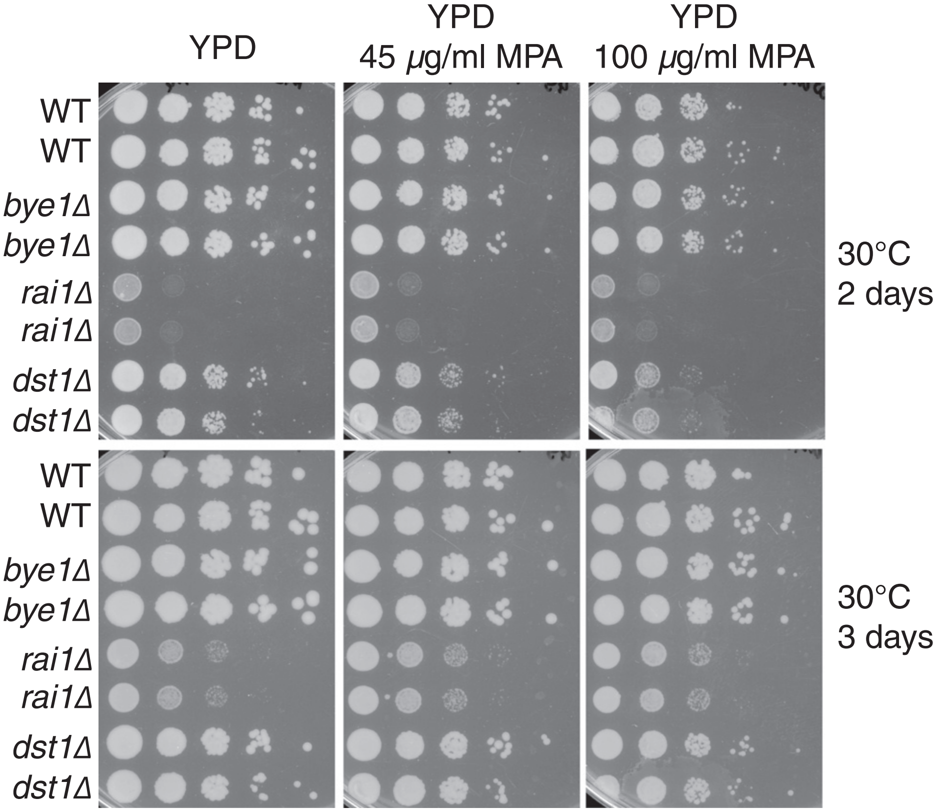

Figure EV2. MPA growth assay for wild-type cells and *bye1Δ*, *rai1Δ*, and *dst1Δ* mutants.

WT, *bye1Δ*, *rai1Δ*, and *dst1Δ* mutants were plated on YPD media containing 0, 45, or 100 μg/ml of the transcription elongation inhibitor mycophenolic acid (MPA). Deletion of the transcription elongation factor *DST1* confers increased sensitivity to MPA at both low (45 μg/ml) and high (100 μg/ml) doses of MPA, consistent with the role of Dst1 as a positive transcription elongation factor. Conversely, *bye1Δ* and *rai1Δ* mutants display decreased sensitivity to MPA, consistent with both *bye1Δ* and *rai1Δ* acting as negative regulators of transcription elongation.

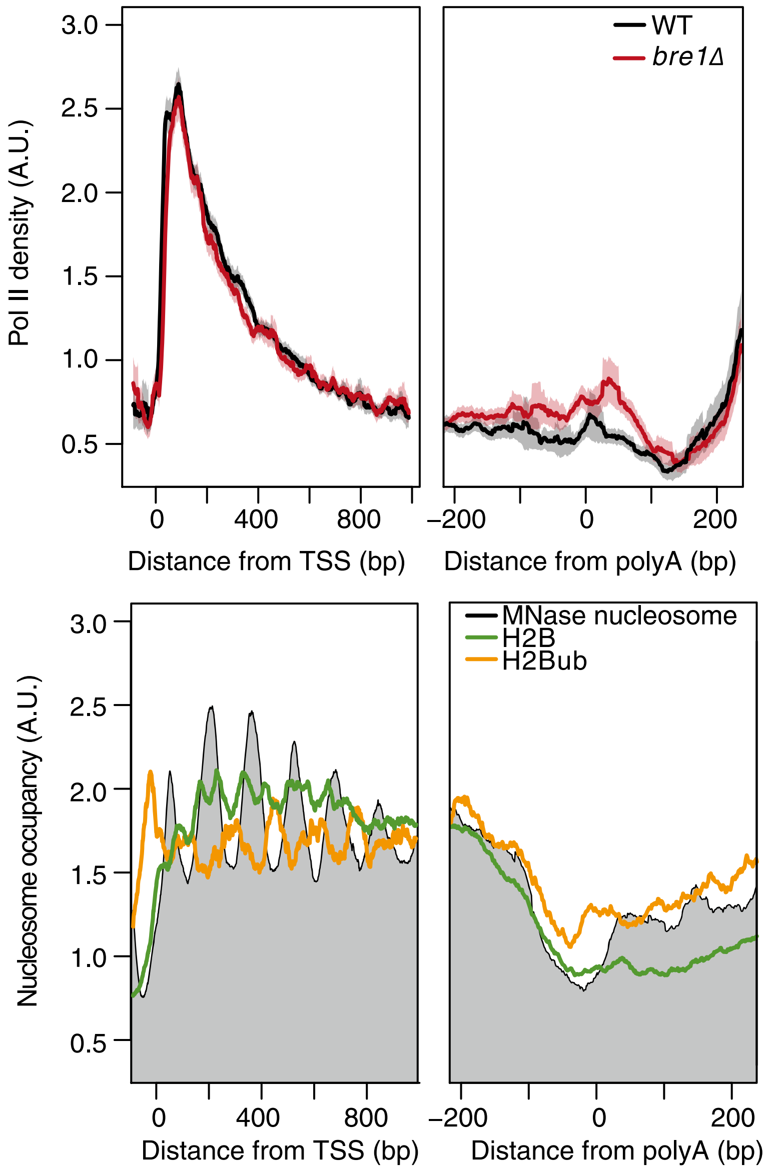

Figure EV3. Bre1 and H2Bub are present on nucleosomes near the polyA site and are not the result of overlap with the plus one nucleosome from neighboring genes.

Top: normalized average NET-seq profiles for WT and *bre1Δ* cells around the transcription start site TSS and polyA of protein-coding genes with non-overlapping genes that do not overlap with another protein-coding gene within 350 base pairs, $n = 1{,}120$. NET-seq reads for each gene are normalized by total reads for each gene in the analyzed region; shaded areas represent the 95% confidence interval. Bottom: average normalized MNase seq (gray) or ChIP-exo for H2B (green), H2B ubiquitylation (H2Bub, gold) around the TSS and polyA sites of the same genes used for the top NET-seq analysis. MNase-seq data were obtained from van Bakel *et al* (2013), and ChIP-exo data were obtained from Rhee *et al* (2014).

© 2017 The Authors
